# The Role of Chemokine Receptor CXCR4 in the Biologic Behavior of Human Soft Tissue Sarcoma

**DOI:** 10.1155/2011/593708

**Published:** 2010-12-22

**Authors:** Roger H. Kim, Benjamin D. L. Li, Quyen D. Chu

**Affiliations:** Louisiana State University Health Sciences Center - Shreveport and Feist-Weiller Cancer Center, Shreveport, LA 71130-3932, USA

## Abstract

The molecular basis of sarcoma remains poorly understood. However, recent studies have begun to uncover some of the molecular pathways involved in sarcomagenesis. The chemokine receptor CXCR4 has been implicated in sarcoma development and has been found to be a prognostic marker for poor clinical outcome. There is growing evidence that overexpression of CXCR4 plays a significant role in development of metastatic disease, especially in directing tumor cells towards the preferential sites of metastases in sarcoma, lung and bone. Although further investigation is necessary to validate these pathways, there is potential for clinical application, particularly in the use of pharmacologic inhibitors of CXCR4 as means of preventing sarcoma metastasis.

## 1. Introduction

Sarcomas are relatively rare tumors of mesenchymal origin, accounting for less than 1% of malignancies [1, 2]. The American Cancer Society (ACS) estimates that there will be 10,500 new cases of soft tissue sarcoma in 2010 [1]. An estimated 3,920 patients will die in 2010 from sarcoma in the US [1]. In addition to their rarity, sarcomas are a heterogeneous group of malignancies, with over 50 different histologic subtypes with highly variable microscopic appearance and clinical behavior [3]. The combination of rarity and diversity has made scientific investigation into the molecular basis of sarcomas challenging [2]. Indeed, even the cell of origin in sarcomas remains unidentified and a subject of controversy [4]. However, recent studies have started to uncover some of the molecular markers and pathways that contribute to human sarcomagenesis [4]. Among these recent discoveries is the role that the chemokine receptor CXCR4 plays in the pathogenesis of several subtypes of sarcoma. In this paper, we will review the literature on the function of CXCR4 in human sarcomagenesis.

## 2. Chemokine Receptor 4 (CXCR4)

Chemokines are 8 to 12 kDa peptides that function in cell differentiation, migration, and trafficking by acting as chemoattractant cytokines [5]. There are four groups of chemokine receptors: C, CC, CXC, and CX3C. Chemokine receptor 4 (CXCR4) is a seven-transmembrane G protein-coupled chemokine receptor [6]. CXCR4 is normally expressed on T-lymphocytes, B-lymphocytes, monocytes, macrophages, neutrophils, eosinophils, in addition to being present in brain, lung, colon, heart, kidney, and liver cells [5]. CXCR4 is also expressed on astrocytes, neuronal cells, and smooth muscle progenitors [5]. CXCR4 is also the chemokine receptor most commonly expressed in tumor cells, with increased expression in melanoma, breast, ovarian, gastric, prostate, colorectal, and lung cancer [7–10]. High levels of CXCR4 have been shown to correlate with the presence of metastatic disease in a wide variety of malignancies, including breast, prostate, lung, colorectal cancer, melanoma, and neuroblastoma [8, 10–16]. CXCR4 has also been demonstrated to be involved in cell migration and invasion, as well as angiogenesis.

The activation of CXCR4 by its ligand, CXCL12, initiates multiple intracellular signaling cascades [5]. CXCL12, also known as stromal cell-derived factor-1 (SDF-1), is a homeostatic chemokine. CXCL12's major function is in regulating hematopoietic cell trafficking and secondary lymphoid tissue architecture. In malignancy, high expression of CXCL12 has been found in lung and bone, tissues that are preferential sites for certain malignancies, such as breast cancer.

## 3. Osteosarcoma

Osteosarcoma, also known as osteogenic sarcoma, is the most common primary bone malignancy [4]. CXCR4 is expressed in 67% of osteosarcomas, with high levels of expression correlating with decreased overall survival, event-free survival, and metastasis-free survival [17]. Survival is only 10% in tumor samples that express CXCR4 mRNA, compared to 90% survival in tumor samples that do not express CXCR4 mRNA. CXCR4 expression level also correlates with the presence of metastasis at diagnosis [17]. Human osteosarcoma cell lines also have been found to express high levels of CXCL12 [17].

Osteosarcoma preferentially metastasizes to lung and bone, tissues with high levels of CXCL12 [10]. Osteosarcoma cells expressing CXCR4 migrate towards a CXCL12 gradient [18]. Adhesion of osteosarcoma to endothelial and bone marrow stromal cells is also promoted by CXCL12. In addition, there is a significant correlation in osteosarcoma between CXCR4 and expression of vascular endothelial growth factor (VEGF), a critical mediator of angiogenesis and tumor proliferation [19].

The role of CXCR4 in osteosarcoma metastasis has been further validated in animal models. The T134 peptide, a CXCR4 inhibitor, was found to prevent the development of lung metastasis after the injection of osteosarcoma cells in a mouse model [18]. In another study, administration of CTCE-9908, also a CXCR4 inhibitor, resulted in a 50% decrease in the number of metastases in mice injected with osteosarcoma cells [20].

## 4. Rhabdomyosarcoma

Rhabdomyosarcoma (RMS) is the most common soft tissue malignancy in children [21]. CXCR4 is highly expressed on the surface of RMS cells, with higher expression in the more clinically aggressive alveolar subtype of RMS compared to the embryonal subtype [21, 22]. High CXCR4 expression also correlates with unfavorable primary sites, advanced stage, marrow involvement, decreased overall survival, and event-free survival in RMS [23].

CXCL12 has no effect on the proliferation or survival of RMS cells, but does stimulate processes related to cell invasion and metastasis [22]. CXCL12 increases adhesion of RMS cells to endothelium. RMS cells also follow a directional chemotaxis towards bone marrow stroma, a CXCL12-rich environment, which may indicate a role of CXCR4 in tendency of RMS to preferentially metastasize to bone marrow [21, 22]. However, CXCL12 did not increase the survival of RMS cells exposed to radiation or chemotherapy, indicating that CXCR4 may not play a role in the development of treatment resistance [24].

## 5. Chondrosarcoma

Chondrosarcoma is the second most common primary bone malignancy, after osteosarcoma [25]. CXCR4 and CXCL12 expressions have been found to be increased in both chondrosarcoma tissue and cell lines [26], with the expression of CXCR4 correlating with tumor grade. CXCR4 signaling regulates the expression of matrix metalloproteinase 1 (MMP1), a marker of chondrosarcoma tissue invasion, metastasis, and poor prognosis [25]. In addition, CXCR4 signaling appears to partially mediate hypoxia-induced increases in MMP1 expression [25].

## 6. Ewing's Sarcoma

Ewing's sarcomas are poorly differentiated tumors and have high metastatic potential [4, 27]. A subset of Ewing's sarcoma tumors and cell lines predominately express CXCR4. High expression of CXCR4 correlates with metastatic Ewing's sarcoma and with poor patient survival [27]. Also, CXCL12 has been demonstrated to be a potent stimulator of invasion by Ewing's sarcoma cells [28].

## 7. Malignant Fibrous Histiocytoma

Malignant fibrous histiocytoma (MFH), also termed as high-grade undifferentiated pleomorphic sarcoma, is one of the highest-grade soft tissue sarcomas [4]. CXCR4 expression has been shown to be upregulated in MFH tumor cell lines [29].

## 8. Other Soft Tissue Sarcomas

In a heterogeneous group of malignant nonround cell tumors, which included synovial sarcoma, malignant peripheral nerve sheath tumor, leiomyosarcoma, MFH, liposarcoma, fibrosarcoma, angiosarcoma, clear cell sarcoma, epithelioid sarcoma, osteosarcoma, and chondrosarcoma, high CXCR4 mRNA expression was an independent predictor of poor prognosis by univariate and Cox multivariate analysis [30]. There was also a significant correlation between CXCR4 and expression of VEGF in this group of soft tissue sarcomas [30].

## 9. CXCR4 Inhibition

Because of the wealth of evidence implicating CXCR4's role in metastatic disease for a variety of malignancies, CXCR4 inhibition has been investigated for its potential for clinical application in cancer therapy. Plerixafor (AMD3100) was initially discovered as an anti-HIV agent and later found to be a potent selective inhibitor of CXCR4 [31]. Recently, it has been utilized in multiple myeloma and non-Hodgkin's lymphoma as a hematopoietic stem cell mobilizer [32]. The use of plerixafor as a chemotherapeutic agent has been suggested for nonhematologic malignancies, including glioblastoma, various gastrointestinal cancers, melanoma, and lung cancer [14, 20, 33, 34].

In regards to CXCR4 inhibition for sarcoma, as mentioned previously, CXCR4 inhibitors have been validated in two animal models of osteosarcoma metastasis. The CXCR4 inhibitors T134 peptide and CTCE-9908 have both been shown to decrease or prevent the development the osteosarcoma metastases in mice [18, 20]. Also, inhibition of CXCR4 by plerixafor resulted in decreased directional cell migration of a rhabdomyosarcoma cell line [21].

## 10. Summary

CXCR4 appears to be a useful prognostic marker for multiple histologic subtypes of soft tissue sarcoma. High expression levels of CXCR4 are correlated with poor outcomes and also predict metastatic disease. There is evidence that CXCR4 and its ligand, CXCL12, play a critical role in the preferential targeting of sarcoma metastases towards lung and bone. Finally, in vivo data indicate the potential of CXCR4 as a target for chemotherapy agents and the possible use of CXCR4 inhibitors in preventing the development of metastasis from sarcomas. Further studies will be necessary to translate this potential into application in the form of clinical trials.
